# Remics: a redescription-based framework for multi-omics analysis

**DOI:** 10.3389/fcell.2026.1738010

**Published:** 2026-03-04

**Authors:** Aritra Bose, Daniel E. Platt, Kahn Rhrissorrakrai, Myson Burch, Aldo Guzmán-Sáenz, Niina Haiminen, Laxmi Parida

**Affiliations:** 1 IBM T.J. Watson Research Center, Yorktown Heights, NY, United States; 2 DAIN Studios, Helsinki, Finland

**Keywords:** biomarker discovery, data mining, disease prediction, genetic epidemiology, multi-omics, networks, statistics

## Abstract

Complex diseases such as cancer are characterized by their intricate etiology, arising from several molecular mechanisms that span multiple omic layers. To obtain insights on disease subtypes, associated biomarkers, and improve prognostic modeling, it is essential to integrate and interpret multi-omics data in a biologically meaningful way. We introduce Remics, a redescription-based framework for multi-omics integration inspired by higher-order statistical representations. Remics leverages higher-order cumulants to identify redescriptions, which are sets of multi-omics features that jointly capture equivalent biological variation across modalities. These feature groups are further analyzed through network representations, multi-omics risk scoring, and biomarker discovery to reveal molecular interactions underlying disease mechanisms. We applied Remics on simulated data as well as multi-omics data of six different cancer types from The Cancer Genome Atlas. We demonstrate that redescription-based integration uncovers functionally coherent cross-omics feature associations and compare them with state-of-the-art approaches. Our results highlight the potential of higher-order multi-omics statistical analysis to advance precision medicine through improved interpretability and discovery of novel molecular relationships.

## 1 Introduction

Since understanding the etiology and pathogenesis of a disease provides the necessary basis for targeted treatment and prevention, the modern challenge for complex disease genetics is to discover how biomarkers from different omics platforms, such as genome, transcriptome, proteome, metabolome, etc., can impact complex disease phenotypes in concert. It is through characterizing and leveraging their interactions that we can elucidate the molecular mechanisms of complex diseases by discovering the associated biomarkers and predict disease outcome and its subtypes. As multi-omics approaches often inherit the challenges from single-omic analysis, this integration is typically fraught with challenges ranging from platform diversity and intrinsic heterogeneity within single omics (Subramanian et al., 2020) to missing data across different omics leading to sparsity (Song et al., 2020), to the varying dimensions for each omics, e.g., genomics and transcriptomics data often being magnitudes larger than metabolomics or proteomics, etc.

Many multi-omics data integration and analysis tools exist which try to address the above problems and derive some means of actionable biological insight. Some integration strategies use feature selection to reduce dimensions or perform early integration of all the omic profiles together in one large matrix (Picard et al., 2021). Recently, deep learning methods have been implemented to reduce dimensionality of the multi-omics matrix after early integration to extract embeddings associated with disease outcome (Chaudhary et al., 2018), while other methods use regularized multiple kernel learning to reduce dimensionality (Speicher and Pfeifer, 2015). However, these approaches often suffer from lack of interpretability. Network based methods have been extensively developed for multi-omics data, including fusion methods such as Similarity Network Fusion (Wang et al., 2014) and multi-layer networks (Lee et al., 2020). These network based approaches use clustering to build upon a patient similarity matrix often applied to partial data sets (Rappoport and Shamir, 2019) and do not provide interactions between multi-omics features for affected patients. Some methods create new common latent representations from multi-omics data with matrix factorizations (Lee and Seung, 1999; Argelaguet et al., 2018; Rohart et al., 2017). One such approach employs multi-omics factor analysis (MOFA), a generalization of principal component analysis (PCA), to integrate multi-omics in an unsupervised manner. However, most of these methods are based on linear multivariate methods that do not take higher-order interactions between the multi-omics variables into account and is targeted towards biomarker discovery and classifying disease subtypes or outcome rather than interactions between features.

Here, we propose Remics, a redescription-based multi-omics analysis tool, which performs redescription mining, leveraging higher-order correlations between different omics features to obtain ensemble meta-features that represents the redescription groups of the individual features from single-omic profiles. Redescription mining (Parida and Ramakrishnan, 2005) is a conceptual clustering method which redefines sets of objects from multiple datasets, situates knowledge gained from one dataset in the context of others, and harnesses high-level abstractions in the form of meta-features thereby recovering subtle cryptic features in the data and exposing novel patterns therein. These meta-features can be used for learning association between multi-omics biomarkers. Remics has two primary components for downstream analyses, after computing the redescription groups: (1) cumulant-based network analysis (CuNA), and (2) cumulant-based risk scores (CuRES). CuNA computes a network by projecting these higher-dimensional interactions and analyzes it to identify hidden interactions in the data, subsequently discovering biomarkers. It also provides an interactive visualizer, which can be used to investigate the network for motifs, clusters, and to identify interactions between multi-omics variables. CuRES computes a single-value estimate of risk of a trait or disease per individual from the redescription groups of multi-omics variables. We applied Remics to simulated data representing multi-omics with varying degrees of correlations among them. To demonstrate how Remics integrates multi-omics features into biologically informative redescription groups, predicts disease status, and discovers latent interactions between variables, we applied it on six cancer datasets with transcriptomic, epigenetic, microRNA (miRNA), and clinical data. We compared Remics with conceptually similar state-of-the-art multi-omics integration methods, such as SNF that uses a fused network of samples across multi-omics data to integrate them with fused variables (Wang et al., 2014), and MOFA, which uses factor analysis to infer a low-dimensional representation across multiple data modalities, capturing global sources of variability (Argelaguet et al., 2018). Multi-omics variables are often correlated at higher-order (any order greater than two) than pairwise, when integrating more than two single-omic datasets. Moreover, due to underlying biological similarities of samples, some outcomes are better explained with multi-way interactions between single-omic variables. Remics enables integration of multi-omics data leveraging higher-order interactions between the variables to provide an informative and interpretable framework of analyzing multi-omics data.

## 2 Materials and methods

### 2.1 Redescription groups

In a dataset with S samples and F features, s∈S are described by a list of features $f_{i}\left(s\right)$ , i∈F . Let there be a group $G_{i}$ associated with each feature, $f_{i}\left(s\right)\in G_{i}$ , where $G_{i}\in R$ . Examples of features in F can include genes, genomic markers, clinical variables, or other variables in multi-omics data. For a given $g_{i}\in G_{i}$ , the set of subjects that have that value is $f_{i}^{-1}\left(g_{i}\right)\subseteq S$ . These features can be binary or continuous variables, where continuous variables can be converted to binary according to a threshold, such as the mean $f_{i}\left(S\right)$ , mapped to 1 if $f_{i}\left(s\right)\geq m\left(f_{i}\left(S\right)\right)$ . Patterns in the data can be described in terms of conjunctions i∧j for i,j∈F such that $f_{i\wedge j}^{-1}\left(g_{i},g_{j}\right)=f_{i}^{-1}\left(g_{i}\right)\cap f_{j}^{-1}\left(g_{j}\right)$ for binary $g_{i},g_{j}$ . This definition can be extended to include not only i,j but also to any combinations of conjunctions subject to the logical algebra of ∧ (e.g., (i∧j)∧(i∧k)=i∧j∧k for i,j,k∈F subject to values $g_{i},g_{j},g_{k}$ . In other words, if there are three features that interact with each other, such as genes PARP1 and BRCA family of genes, which are strongly related in many cancers causing tumor cell death, this interaction will be represented as $f_{PARP1\wedge BRCA1\wedge BRCA2}^{-1}\left(PARP1=1,BRCA1=1,BRCA2=1\right)$ . Such combinations of conjunctions i that have more or less members $f_{i}^{-1}\left(a\right)$ than expected by chance are called *patterns*. Binomial and other tests of the significance of patterns can be dominated by lower-order correlations among the variables in a pattern.

Definition 1 *Two distinct patterns that yield the same subsets of subjects, e.g.,* $f_{i}^{-1}\left(g\right)=f_{j}^{-1}\left(g\right)$ *, are called “redescriptions”.*

If conjunctions yield a form such as A∩B=B , then it may be deduced that B⊂A , and the conditions yielding A and B satisfy b⇒a (see Supplementary Note for details). In other words, redescriptions can reveal logical relationships among features. Such relationships may reflect the underlying biological pathways reflected in these connected phenotype patterns. Therefore, each of these patterns i specifies a phenotype, which may be associated with genotypes or other omic data using standard methods. Redescription groups are often generated combinatorially by measuring significant associations among features. However, if those groups are selected from highly correlated variables, then it may be difficult to extract distinct interactions among these variables. We solve this problem by testing redescription groups using Fisher permutation test. This identifies whether one factor significantly affects the relationships between other factors (Karisani et al., 2022).

#### 2.1.1 Redescriptions via cumulants

An intuitive way to find redescription groups is to compute cumulants (Parida and Ramakrishnan, 2005; Platt D. et al., 2024; Karisani et al., 2022; Bose et al., 2021; Bose et al., 2023; Bose et al., 2026), which identify logical relationships between the features defining the patterns. Multiple patterns capture the same set of samples, and thus find the redescription groups (Definition 1). Since most of the biomarkers in large biobanks are strongly correlated, we need to factor out those strong lower-order correlations from higher order associations marking distinct groups of individuals differentiating sub-types of the disease. Cumulants, in simple terms, are measures of the interaction of random variables in a probability distribution. If f(X) is a function of any random variable X with outcome $\left\{x_{i}\right\}$ , then its expectation is given as $E\left[f\left(X\right)\right]=\sum_{i}p_{i}f\left(x_{i}\right)$ . The first order cumulant is defined as the mean of X , defined as $\sum_{i}p_{i}x_{i}$ and thus the higher order moments $<X^{d}>=\sum_{i}p_{i}x_{i}^{d}$ . This is formalized as a moment generating function,

$$M\left(\lambda \right)=\overset{\infty }{\underset{i=0}{\sum }}\frac{\lambda^{i}}{i!}<X^{d}>$$

Thus, the moments of X can be generated as the terms of coefficients of the Taylor expansion of M(λ) . The cumulant generating function is nothing but the logarithm of M(λ) , defined as log⁡M(λ) . The first four orders of cumulants are known as mean, variance, skewness, and kurtosis (a detailed introduction to cumulants are in the Supplementary Note).

### 2.2 Remics

Remics, or redescription-based multi-omics analysis, computes redescription groups via cumulants, here calculated using the Julia package Cumulants.jl, to capture higher-order interactions between multi-omics variables using multidimensional tensors (Domino et al., 2018). Remics takes as input a real-valued matrix in which rows correspond to samples and columns represent molecular features from one or more omics layers (e.g., transcriptomic, epigenomic, proteomic, metabolomic, etc.). Multi-omics data may be integrated using early fusion by concatenating feature matrices across modalities, or using late fusion by supplying a preprocessed composite representation. While both strategies are supported, early fusion facilitates more direct interpretability of the resulting redescription groups. The input matrix is normalized prior to computing higher-order cumulants; alternatively, users may provide appropriately normalized data. Remics makes no modality-specific modeling assumptions beyond this normalization step.

Algorithm 1 **Remics: Redescription-based multi-omics analysis.**

**Input:** A matrix $A\in R^{m\times n}$ , where m is the number of individuals and n is the number of multi-omics features; order of computation, d

**Output:** Set $C^{\prime }$ of statistically significant redescription groups, $|C^{\prime }|=k$ ; matrix $M\in R^{n\times k}$ , with each sample having k -dimensional vectors representing redescription meta-features
 1: $\bar{A}=A-1_{m}\mu^{⊤}$ , where $\mu_{j}=\frac{1}{m}\sum_{i=1}^{m}A_{ij},\;j=1,2,\ldots ,n$

 2: Compute G ’s (Supplementary Equation 2 in Supplementary Note) to identify higher-order cumulants from $\bar{A}$ for order d

 3: Perform Fisher permutation tests and obtain $C\in R^{m\times n^{d}}$ along with their parameters of statistical significance, $z=\frac{C-\mu }{\sigma }$ , p=P(Z≥z) , where μ : expected value or mean under null hypothesis, and σ : standard deviation
 4: Obtain $C^{\prime }=\left\{c_{i}\in C|p_{i}<0.05\right\}$ of statistically significant redescription groups of size k

 5: Obtain the cumulant loadings for $C^{\prime }$ from G (Supplementary Equation 2 in Supplementary Note) in matrix $M\in R^{n\times k}$

 6: Perform network analysis using CuNA (Algorithm 2) using $C^{\prime }$

 7: Compute risk score estimates per sample using CuRES (Algorithm 3) using M

**FIGURE 1:**
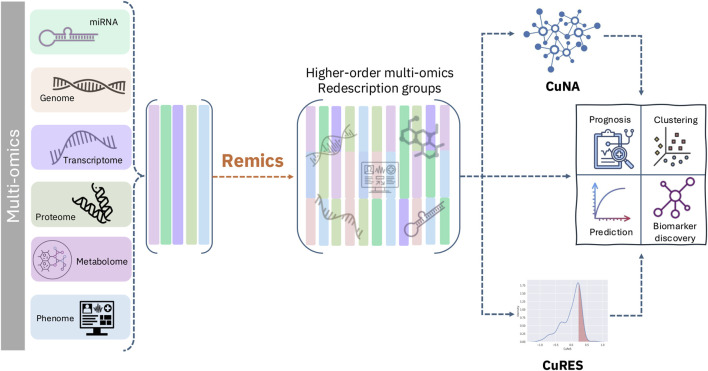
Remics framework demonstrating ingestion of multi-omics data and computation of redescription groups by cumulants and subsequently computing CuNA (Algorithm 2) and CuRES (Algorithm 3).

The cumulants lead to meta-features which are a combination of multi-omics features. It uses this latent interaction space between variables to extract meaningful combinations between the different omics features and analyze them (Figure 1, details in Algorithm 1).

Algorithm 2 **CuNA: Cumulant-based Network Analysis.**

**Input:** Set $C^{\prime }$ of statistically significant redescription groups, $|C^{\prime }|=k$

**Output:** (i) K Communities of interactions between the multi-omics variables; (ii) ranking of nodes, V sorted by their relative importance in the network; (iii) p-values, p , and weights, w , of the edge interactions;
 1: **FOR** all pairs of features ( $f_{i}$ , $f_{j}$ ) in g redescription groups $\left(g\in C^{\prime }\right)$ :
 2: $s_{ij}$ : number of times ( $f_{i}$ , $f_{j}$ ) appear together in g

 3: Build a network, G=(V,E) where vertices are features $f_{i}$ and $f_{j}$ , $f_{i}\in V$ and |V|=n and edge $e_{ij}\in E$ with weight $w\left(e_{ij}\right)=s_{i,j}$ .
 4: **FOR** all g groups of features:
 5:  **FOR** all $\left(f_{i},f_{j}\right)$ pair of k2 features:
 6:   Build a contingency table $N_{i,j}$ = $n_{i,j}$ , $n_{*,j}$ , $n_{i,*},n_{*,*}$ .
 7:   Obtain p-value $p_{i,j}$ Fisher’s exact test on $N_{i,j}$

 8:   **IF** $p_{i,j}<0.05$

 9: $E\left[\cup \right]e_{i,j}$

 10: $V\cup \left(f_{i},f_{j}\right)$

 11:   **END IF**
 12:  **END FOR**
 13: **END FOR**
 14: Perform community detection using any method of choice (Yang et al., 2016) and obtain K communities.
 15: Obtain the ranking of nodes in the network $\left\{f_{1},f_{2},\ldots ,f_{n}\right\}$ using centrality analysis aggregation.
 16: Obtain (p,w)∀e where e∈E .

### 2.3 CuNA

Cumulant-based network analysis (CuNA) computes a network with statistically significant connections between any pair of features in $M\in R^{n\times k}$ (from Algorithm 1). It analyzes the network by extracting communities, ranks the features based on their relative importance in the network, and obtains insights on molecular underpinnings of clustered multi-omics features. Manual inspection of the entire network can be challenging when the network is dense. To aid the parsing of results obtained from CuNA analysis, we developed an interactive web-tool to display relevant collections of sub-graphs or communities and highlight their common edges allowing the user to query the network. An outline and details of this algorithm are present in Algorithm 2 with further details on the ranking procedure and the visualizer in the Supplementary Note.

#### 2.3.1 Parameter selection and robustness

CuNA employs a user-defined p− value threshold to control edge inclusion, reflecting the problem-specific nature of multi-omics association structure. We recommend evaluating thresholds over a broad range (e.g., $p\in \left[10^{-2},10^{-16}\right]$ with step sizes of $10^{-2}$ or $10^{-4}$ ) and assessing network stability by examining node rankings, community assignments, and global network statistics across this range. While edge density varies with stringency, high-centrality nodes and major communities remain stable over wide range of thresholds, indicating that CuNA captures higher-order interaction structure rather than threshold-specific artifacts. CuNA uses Fisher’s exact test, whose conservative behavior in sparse, high-dimensional settings provide additional protection against false-positive edge inflation.

Algorithm 3 **CuRES: Cumulant-based Risk Scores.**

**Input:** A matrix $M\in R^{m\times k}$ , where n is the number of individuals and k is the number of statistically significant redescription groups; outcome, $y\in R^{m}$ .
 **Output:** A vector $s\in R^{n}$ representing CuRES.
 1: Split M into $M_{tr}$ , training and $M_{te}$ , testing data randomly.
 2: Solve $y=M_{tr}\beta$ where $\beta \in R^{k}$ and obtain the effect size, $\hat{\beta }$ .
 3: $s=\sum_{i}^{K}M_{{te}_{i}}\hat{\beta_{i}}$ where $M_{{te}_{i}}$ is a column of the matrix $M_{te}$ representing the $i^{th}$ redescription group.

### 2.4 CuRES

Remics has capabilities to compute an individual assessment of risk for a trait or disease from multi-omics data, called CuRES, from the redescription groups. It takes significant groups, in the form of meta-features and their corresponding cumulant loadings per individual, and splits the data into train and test sets. It fits a generalized linear model on the training data, learns the effect sizes, and then computes an aggregate sum of the meta-feature values per individuals in the test set weighted by the learned effect sizes. This creates a vector of scores per individual that is significantly associated with the target trait or the incidence of the disease. In its mathematical form, the estimated CuRES, $\hat{S}$ is obtained as the sum across k meta-features, weighted by their weights or coefficient of the linear model, ${\hat{\beta }}_{j}$ .

$$\hat{S}=\overset{k}{\underset{j=1}{\sum }}M_{j}{\hat{\beta }}_{j}$$

CuRES can be generalized to any trait or disease, providing a predisposition or risk estimate per sample for that trait computed from a holistic multi-omics perspective (details in Algorithm 3).

### 2.5 Data

#### 2.5.1 Simulation studies

We designed a multi-omics simulator integrating phenotypes, genotypes, and gene expression levels (Platt D. E. et al., 2024). To handle the integration of different omics data we started with a multivariate distribution

$$f\left(x\right)d^{d}x=\sqrt{\frac{\det\left(A\right)}{{\left(2\pi \right)}^{d}}}\exp\left(-\frac{1}{2}{\left(x-\mu \right)}^{T}A\left(x-\mu \right)\right)d^{d}x$$

Components of x were identified as phenotypic (binary, which may include environmental conditions as well), single nucleotide polymorphisms (SNPs) (pairs of binary alleles, one for each of the chromosome pairs), or gene expression (float). Covariances $A^{-1}$ were specified in terms of $A=\sigma \text{cor}\left(x,x^{T}\right)\sigma$ where the σ is a diagonal matrix with values representing the spread of the variates, and $\text{cor}\left(x,x^{T}\right)$ is specified to yield correlations among phenotypes, alleles between each pair of chromosomes representing Hardy-Weinberg disequilibrium, and among gene expression levels reflecting co-regulation among pathways (more details in Supplementary Material). We simulated three different scenarios for 1,000 samples and 30 features (10 phenotypes, 10 SNPs, and 10 genes with varying expression levels) to demonstrate Remics’ ability to identify genotype-phenotype interactions with the highest Pearson correlation coefficient $\left(r^{2}\right)$ and its robustness in the presence of false positives while correcting for spurious associations. These scenarios with varying correlations were: (i) an extreme case where only a few features among the genes, SNPs, and phenotypes were highly correlated with each other (Figure 3); (ii) an average case where many of the features were moderately correlated with each other (Supplementary Figures S1, S2); (iii) a sanity check with completely uncorrelated features, therefore, the resulting correlation matrix being equal to an identity matrix.

#### 2.5.2 TCGA multi-omics datasets

**FIGURE 2:**
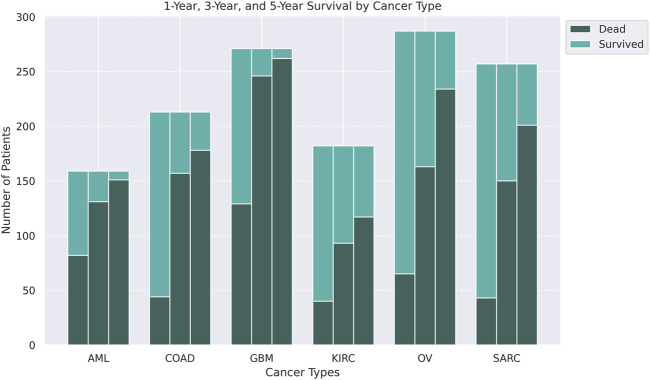
Stacked barplots of the number of patients along with their status information (dead or survived) for 1-, 3-, or 5-year survival (left to right) for six cancers, AML, COAD, GBM, KIRC, OV, and SARC.

We utilized six processed, benchmark multi-omics datasets from Rappoport et al. (Rappoport and Shamir, 2018) that were derived from The Cancer Genome Atlas (TCGA). Each dataset, which contains miRNA expression, gene expression, and DNA methylation data, represents primary tumor samples from a different cancer: acute myeloid leukemia (AML), colon adenocarcinoma (COAD), glioblastoma multiforme (GBM), kidney renal cell carcinoma (KIRC), ovarian serious cystadenocarcinoma (OV), and sarcoma (SARC). Data was downloaded from http://acgt.cs.tau.ac.il/multi_omic_benchmark/download.html. In summary, these pre-processed datasets excluded patients and features with >20% missing values with remaining missing values imputed using k-nearest neighbor imputation, as well as filtering methylation data for the top 5,000 features by variance. For each dataset, we performed orthogonal matching pursuit (OMP) (Tropp and Gilbert, 2007) using *scikit-learn*’s OrthogonalMatchingPursuit function to select 387 gene expression features, 95 DNA methylation features, and 20 miRNA features (preserving their proportions from the quality controlled data) for all downstream analyses. We processed the “survival” outcome in the data, to create three binary outcomes, namely, “1-year”, “3-year”, and “5-year” survival, to perform downstream prediction and network analysis. The number of overlapping samples across all the cancer types, as well as their number of patients dead or surviving are shown in Figure 2.

### 2.6 Analysis

#### 2.6.1 Classification

We performed classification analysis using CuRES on the cumulant loadings for those significant cumulants observed in $C^{\prime }$ in Algorithm 1. We used different thresholds of significance to filter our results for the best performing threshold by evaluating the results from 12 p -values over the range $p=\left[0.01,10^{-24}\right]$ decremented by $10^{-2}$ . We used LogisticRegression classifier with $L_{1}$ penalty from scikit-learn (Pedregosa et al., 2011) package in Python for each classification task. We used age and gender as confounding variables for the regression analysis. We performed five-fold cross validation with hyperparameter tuning using the GridSearchCV function.

#### 2.6.2 Networks

To test the significance of CuNA, we evaluated the interactions over the same range of p -values as in the classification analysis. The width of the edges corresponds to the number of times a pair of nodes appeared together in the redescription groups and reflects their pairwise affinity. The nodes were ranked in the order of their importance computed as an aggregate score of mean of the ranks in different network centrality measures (see Supplementary Note for more details). We used the networkx package (Hagberg et al., 2008) in Python to perform all network analyses including community detection using greedy modularity method.

## 3 Results

### 3.1 Simulation studies

#### 3.1.1 Network analysis

We applied Remics on different simulation scenarios varying from easy to complex interactions between genes, SNPs, and phenotypes.

**FIGURE 3:**
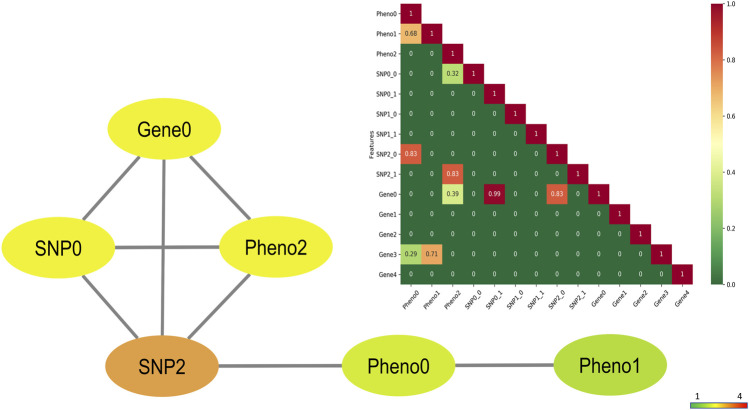
CuNA network of the simulated variables with few highly correlated features. The nodes are colored by degrees (darker colors have higher degree). The correlation matrix of the 11 variables are shown in the inset with color gradient corresponding to the feature correlation. The features are organized in the matrix in the following order: 3 phenotypes, 2 genotypes (two allele each), and 5 genes with the maximum correlation of 0.99 between allele 1 of *SNP0* and *Gene0* shown in red.

In the first scenario of 11 variables, only a few interactions such as (*Gene0*, *SNP0*), (*Gene0*, *SNP2*), and (*Pheno0*, *Pheno1*) had correlation $r^{2}$ of 0.9, 0.8, and 0.6, respectively. We found the CuNA-projected higher-order interactions captured these highly correlated variables (Figure 3) within three communities from the network, mimicking the spiked-in correlations: {*Gene0*, *SNP2*, *Pheno2*}, {*SNP0*}, and {*Pheno0*, *Pheno1*}. Increasing the complexity of these interactions by involving more samples (varying from 100 to 1,000) as well as more variables (varying from 10 to 30), we found that CuNA accurately found the interacting variables and the communities reflected the clusters of the highly correlated variables together (Supplementary Figures S1, S2). Thus, CuNA captures the communities accurately as reflected in the network (Figure 3) as well as the original correlations that were input to Algorithm 1.

#### 3.1.2 Prediction with CuRES

We assigned the phenotype *Pheno0* as the target variable and considered the rest of the 10 variables as part of the data matrix and computed CuRES (Supplementary Figure S3). We observed that the net reclassification index (NRI), which is computed as the difference in classification accuracy when including CuRES as a variable in the data matrix versus the original data matrix without CuRES, was 1% with a prediction accuracy (measured by the ${\text{F}}_{1}$ score) of 84.7% on held out data after five-fold cross validation with a logistic regression model. When extended to 30 variables and considering *Pheno5* as the target variable, we found that CuRES improved prediction performance by as much as 16% with perfect classification. We note that the effect CuRES has on the NRI varies with the correlation between the simulated variables and number of samples.

### 3.2 Analyzing TCGA mult-omics cancer data

We analyzed six cancer types from TCGA using Remics on the 500 top selected features and computed redescription groups of order 3. For each cancer type, we performed an early integration of the selected transcriptomic, DNA methylation, and miRNA features, after normalization, for each outcome (“1-year”, “3-year”, and “5-year”) based on the survival information. We observed a significant expansion of the feature set for each cancer, with the number of significant (p<0.01) redescription groups yielding up to approximately 700,000 meta-features (Supplementary Figure S4).

#### 3.2.1 Network analysis

**FIGURE 4:**
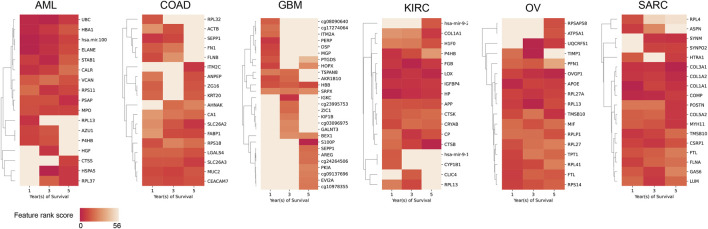
Heatmap of feature rank scores for top features per year of survival for each cancer type. Cells are shaded according to their aggregated feature rank score (darker color signifies higher rank).

We applied CuNA (Algorithm 2) after deriving the statistically significant redescription groups from Remics. We filtered edges for statistical significance (p<0.01) , and obtained varying number of vertices, represented as multi-omics features (AML: 236; COAD: 279, GBM: 375; KIRC: 259; OV: 338; SARC: 319) and edges (Supplementary Figure S5). We obtained the highest-ranked features as well as the four top-ranked edges based on their weight for each cancer type from the networks using our network centrality-based ranking algorithm (Figure 4; Table 1; see Supplementary Table S1 for details).

**TABLE 1:** Top ranked nodes (multi-omics features) and edges (interactions between features) for each cancer type across three outcomes using the network-centrality based ranking algorithm and the edge weight, respectively.

Cancer type	Multi-omics features (nodes)	Interactions (edges)
AML	{UBC, hsa-mir-100, ELANE, HBA1, STAB1}	(ELANE, STAB1), (hsa-mir-100, STAB1), (HSPA5, CTSD), (FTL, UBC)
COAD	{MUC2, SLC26A3, CEACAM7, SLC26A2, FABP1, AZU1}	(TAGLN, ACTG2), (TAGLN, FLNA), (DES, ACTG2)
GBM	{SRPX, HBB}	(AKR1B10, HBB), (AKR1B10, STON1)
KIRC	{LOX, HP, FGB, IGFBP4, CTSB, CP}	(LOX, FGB),(FGB, CP), (COL3A1, COL1A1)
OV	{OVGP1, APOE, FTL, RPL13}	(DLK1, MEST), (DLK1, hsa-mir-891a), (OVGP1, APOE)
SARC	{COL3A1, COMP, COL1A1, COL1A2}	(APOD, MPZ), (PYGM, HSP90B1),(LAMP1, GAS6)

We performed community detection on the network generated by CuNA (Algorithm 2) and obtained communities of multi-omics features. To visualize how the communities interact within and between each other, we projected the networks into our interactive visualizer and selected community subgroups to reveal interactions between ribosomal protein gene network in OV, enriched transcription factor NFE2L2 in AML, DNA methylation interactions in GBM, etc. (Supplementary Figures S6–S8). We evaluated the detected communities for enrichment of biological pathways using the MSigDB Hallmark collection (v2024.1) (Liberzon et al., 2015). Over-represented pathways were identified in each community for every survival year (Supplementary Figure S9). This analysis revealed both well-established and emerging pathway associations that may point to new avenues of investigation. For instance, the *MYC Targets* pathway was significantly enriched across multiple time points in AML and OV, consistent with prior reports of its involvement in these cancers (Ohanian et al., 2019; Call et al., 2020; Ju et al., 2018). Pathways linked to cell cycle regulation—such as E2F targets, G2M checkpoint, and epithelial–mesenchymal transition—were also recurrently enriched across several cancer types and survival years. Additionally, we observed enrichment of hypoxia-related pathways in COAD communities, aligning with growing evidence of hypoxia’s contribution to colorectal cancer progression (Fletcher et al., 2022).

#### 3.2.2 Disease prediction with CuRES

**FIGURE 5:**
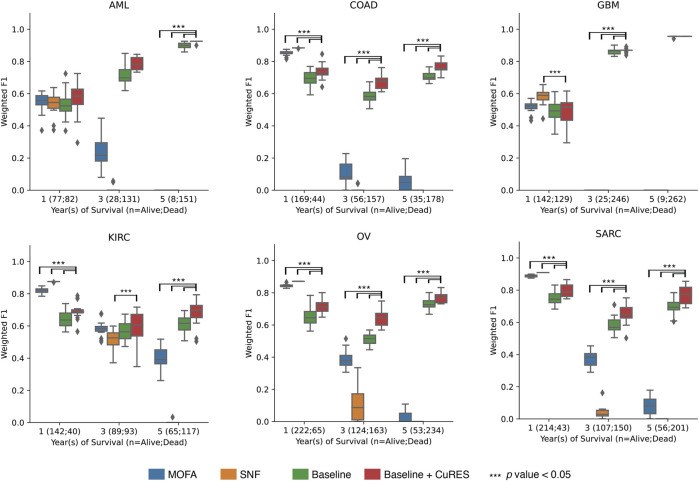
Box and whisker plot showing the weighted $F_{1}$ scores for CuRES, compared with baseline, MOFA, and SNF. Statistically significant (*p* value <0.05 ) with respect to Baseline + CuRES is show in bar above respective boxplot pair.

**FIGURE 6:**
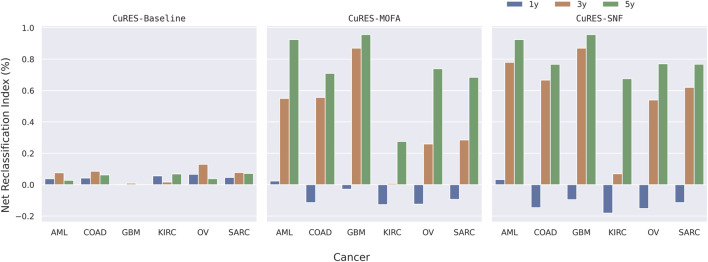
Net Reclassification Index (NRI) in percentage between Baseline + CuRES, Baseline, MOFA, and SNF.

For each cancer type, we performed early integration for multi-omics variables after normalization and used it to serve as the baseline for our experiments. We computed CuRES (Algorithm 3) on the three survival outcomes and on the statistically significant redescription groups obtained from Algorithm 1. We added the CuRES vector to the baseline to check for a change in outcome prediction. To compare with state-of-the-art multi-omics integration methods MOFA (Argelaguet et al., 2018) and SNF (Wang et al., 2014), we applied them on integrated multi-omics data and compared the weighted $F_{1}$ score. To ensure a fair comparison, CuRES, MOFA, and SNF, all were applied to the same training data and evaluated on the same held-out test data. We performed experiments with bootstrapping (n = 20) to obtain the confidence intervals for the weighted $F_{1}$ score. We observed CuRES, when considered as a covariate with the integrated multi-omics data, consistently improves upon the baseline across all the cancers. In most cases CuRES performs better than MOFA and SNF for predicting the “3-year” and “5-year” outcomes (Figure 5). MOFA and SNF both perform better than CuRES when predicting the “1-year” outcome for most cancers where there exists a heavy class imbalance between the number of dead to the number of surviving patients. CuRES had a mean net reclassification index (NRI) of approximately 4% (with baseline), −7% (with MOFA), −10% (with SNF) for the “1-year” outcome; 6% (with baseline), 42% (with MOFA), 59% (with SNF) for the “3-year” outcome; and 4% (with baseline), 71% (with MOFA), 81% (with SNF) for the “5-year” outcome (Figure 6).

### 3.3 Complexity analysis

At the heart of Remics is the computation of cumulants or higher-order moments between features. The computational complexity and resource usage increases exponentially by a factor of $n^{d}$ , where d is the order of the cumulants and n is the set of variables. Thus, overall computational complexity becomes $O\left(mn^{d}\right)$ where m is the number of samples (Domino et al., 2018). Furthermore, the space requirement for computing higher-order cumulants is more challenging. We performed an in-depth complexity analysis using the TCGA data with 1,000 samples and observed a requirement of 100 GB memory to compute sixth-order cumulants with only 50 features (Supplementary Figure S10). Thus, the computation is intractable for very large sets of variables. Although, in practice, it is rare to compute the cumulants beyond fifth order, and the time and memory overhead are significantly less when computing third order cumulants with 100 or fewer features. For the multi-omics analyses done here, the highest memory requirement for Remics was approximately 94 GB, taking 45 min to compute third order cumulants for 271 samples of GBM and 500 features.

## 4 Discussion

Remics, introduced here, is a topology driven, interpretable, integrative multi-omics analysis that supports tasks such as network analysis, biomarker discovery, and disease prediction. Rather than assuming direct regulatory or mechanistic relationship between molecular layers, Remics identifies sets of features that exhibit statistically equivalent variation across samples, capturing higher-order cross-omic structure in a data-driven manner. It builds on the concept of redescription mining (Parida and Ramakrishnan, 2005; Ramakrishnan and Zaki, 2009; Karisani et al., 2022; Platt D. et al., 2024) to uncover multi-omics feature groups that jointly describe shared biological heterogeneity. Remics offers both sample-level and feature-level analyses with CuRES and CuNA, respectively. CuRES provides an individual-level measure of disease liability or risk by leveraging the multi-omics structure uncovered by Remics. Rather than relying on individual molecular features, CuRES represents each patient using redescription groups, which are interpretable multi-omics, meta-features that capture statistically equivalent variation across samples. With the increasing availability of large, EHR-linked biobanks and sequencing technologies, this framework enables a holistic and extensible summary of disease risk that integrates multi-omics and multi-modal data within a unified representation. The risk score estimates that are computed from meta-features in Remics provide a logical extension to the polygenic risk score in genomics, which has found widespread usage in research and medicine (Choi et al., 2020). However, comparing CuRES with PRS is beyond the scope of this work.

Applying CuRES to TCGA cancer cohorts across multiple survival data, we observed that CuRES outperforms established multi-omics integration methods such as MOFA and SNF, in predicting “3-year” and “5-year” outcomes. In contrast, under conditions of severe class imbalance (>3.5:1 between deceased and surviving patients), which most frequently arise in “1-year” outcome prediction, MOFA and SNF tend to achieve better weighted $F_{1}$ scores by favoring the majority class. In these settings, CuRES yields more balanced precision–recall trade-offs, reflecting its emphasis on stable, cross-omics structure rather than majority-class optimization. We therefore recommend interpreting short-term outcome predictions using class-specific metrics (e.g., recall for early mortality) in conjunction with weighted $F_{1}$ , particularly when therapeutic decision-making is time sensitive. Importantly, CuRES generalizes well to unseen, held-out data and supports transfer learning in multi-omics analysis by providing pretrained models and summary statistics for redescription groups, enabling reuse across cohorts and outcome horizons.

Complementarily, CuNA embeds significant higher-order interactions in the form of redescription groups to a network of multi-omics features. The edge weight between a pair of features in CuNA signifies the number of times they were together in higher-order redescription groups. Thus, interactions with highest weights indicate their relative importance in explaining underlying biological functions of the disease and can be seen as a pattern discovery mechanism in multi-omics data. The interactions found by CuNA were validated using the IntAct database of molecular interactions (Del Toro et al., 2022), such as the interactions between ELANE, HSPA5, FLT, UBC, etc. in AML, which are connected by heparin, an anticoagulant in blood and is a known treatment for AML (Kovacsovics et al., 2018). Another interaction that highlighted the underlying biological function was between the TAGLN and ACTG2 genes, which were connected by CTFR in IntAct. TAGLN has a long history of association with COAD (Zhou et al., 2016) and CFTR with cystic fibrosis also has associations with COAD (Scott et al., 2020). In KIRC, the interaction between LOX and COL1A1 were also independently validated in literature (Di Stefano et al., 2016).

CuNA was able to find implicit and explicit connections to the respective cancer types (see Supplementary Note for other validated interactions). For example, most of the top-ranked features for AML, such as UBC, hsa-mir-100, etc. have been shown to be associated with AML. The UBC (Ubiquitin C) ligase determines AML growth and susceptibility to histone deacetylase inhibitors (Khateb et al., 2021), while the miRNA hsa-mir-100 regulates cell differentiation and survival by targeting RBSP3, a phosphatase-like tumor suppressor in AML (Zheng et al., 2012). These connections are found in all of the cancer types studied. Loss of function mutations in MUC2 has been shown to increase colon cancer tumor progression (Hsu et al., 2017; Betge et al., 2016). SRPX is being investigated as a biomarker for GBM (Ampudia-Mesias et al., 2022). OVGP1 expression has been associated with OV (Wu et al., 2016), and COL1A1-PDGFB gain of function drives tumor growth in SARC (Abbott et al., 2006).

Despite all its prowess with multi-omics data, Remics is sensitive to the early integration pitfalls of multi-omics data as discussed in this review (Subramanian et al., 2020). Most of the multi-omics methods are often plagued by this issue (Picard et al., 2021) and thus use different strategies, such as variable selection or latent space analysis, to work around the bottleneck. While Remics’ main limitation is scalability in massive datasets for orders d≥3 (Supplementary Figure S10) owing to the technical limitations of current computational devices and thus leading to the use of mitigating techniques, such as feature selection. This reduction of complexity is not a required component of Remics, but rather a concession to available computational hardware. While such dimensionality reduction techniques are commonplace in biological data analysis, we have endeavored to perform an unbiased feature selection of sufficient size to, some degree, mitigate concerns of skewing results towards known cancer driver genes or pathways. As computing advances such dimensionality reduction methods may become obsolete, for example, by using approximate cumulants from tensor decomposition techniques (Morton and Lim, 2009; Domino et al., 2018) or developing quantum computing algorithms for computing cumulants (Bose et al., 2026) or tensor decomposition (Burch et al., 2025), thereby fulfilling the promise of faster, scalable computation to higher-orders, thus allowing the full promise of Remics to be realized. Furthermore, as cumulants are widely used in many fields of research such as economics, physics, etc., a speed-up on cumulant computation would have wider implications beyond healthcare and biology.

## 5 Conclusion

Associations between multi-omics variables can be complex and often confounded by environmental factors. We propose a framework, Remics, to identify associations with more granularity than a standard case-control association study while performing tasks such as biomarker discovery and prediction of traits in complex diseases. We demonstrate that Remics captures true associations by validating it on simulated data as obtained from the multi-omics simulator (Platt D. E. et al., 2024). We demonstrated CuNA’s application in multiple diseases such as AML, COAD, GBM, KIRC, OV, and SARC, where it was able to find functionally relevant biomarkers, predict outcomes, and perform better than the state-of-the-art methods such as MOFA and SNF. Lastly, we show that the CuRES module can be a useful predictor of the disease state and help to understand an individual’s risk of a disease based on multi-omics and multi-modal variables ranging from imaging, transcriptomics, proteomics, metabolomics, etc., rather than just on genomics. Remics provides an exciting opportunity to decode phenotypic and genotypic diversity and discover biomarkers associated with various manifestations of complex diseases, paving the way for accelerated personalized medicine.

## Data Availability

Publicly available datasets were analyzed in this study. This data can be found here: https://portal.gdc.cancer.gov/.
