# Supplementary material for: Remics: a redescription-based framework for multi-omics analysis
Source: Front Cell Dev Biol. 2026 Mar 4;14:1738010. doi: 10.3389/fcell.2026.1738010 (PMC12996823; doi:10.3389/fcell.2026.1738010)
Supplement: Supplementary file 1 [file DataSheet1.pdf]

Remics: A Redescription-based Framework for Multi-Omics Analysis

Aritra Bose[1], Daniel E. Platt[1], Kahn Rhrissorrakrai[1], Myson Burch[1], Aldo Guzmán-Sáenz[1], Niina Haiminen[2], and Laxmi Parida[1,*]

[1]IBM T.J Watson Research Center, Yorktown Heights, NY, USA
[2]DAIN Studios, Helsinki, Finland
[*]Corresponding authors: parida@us.ibm.com

Supplementary Note

1 Methods

`Remics` builds upon cumulants which form higher-order interactions between variables with underlying distinct group of individuals whose pattern memberships give hints to shared disease processes. These variables result in "redescription" clusters which are processed further downstream.

1.1 Cumulants

Correlation expansions emerge naturally as a unique factorization of correlations into products of independent groups of factors [4], and emerges naturally in statistical mechanics quantum field theory, expressed as connected Feynman diagrams and as a set of "one-particle-irreducible" (1PI) diagrams [2]. Such results also connect naturally in statistics of large deviations through Cramér's theorem [5], which also connects to the notion of "effective actions" from quantum field theory. Their generating functions satisfy useful set partition relationships, and have been a part of traditional statistics for some time [3].

This factorization is represented by a moment generating function

$$\begin{aligned}\mathbb{E}\left[\exp\left(\sum_j F_j J_j\right)\right] &= A + \sum_l J_l G_l + \frac{1}{2!}\sum_{ll'} J_l J_{l'} G_{ll'} + \\ &\quad \frac{1}{3!}\sum_{ll'l''} J_l J_{l'} J_{l''} G_{ll'l''} + \frac{1}{4!}\sum_{ll'l''l'''} J_l J_{l'} J_{l''} J_{l'''} G_{ll'l''l'''} + \cdots \\ &= \exp\left(\sum_l J_l K_l + \frac{1}{2!}\sum_{ll'} J_l J_{l'} K_{ll'} + \frac{1}{3!}\sum_{ll'l''} J_l J_{l'} J_{l''} K_{ll'l''} + \right. \\ &\quad \left. \frac{1}{4!}\sum_{ll'l''l'''} J_l J_{l'} J_{l''} J_{l'''} K_{ll'l''l'''} + \cdots \right)\end{aligned} \tag{1}$$

where the F_j are features indexed by j (defined in Algorithm 2), the G's represent moments, A is a constant offset (unity in this case) defined by $J = 0$, and the K's represent higher order

cumulants, e.g. $G_{ij} = E(x_i x_j)$ and $G_{ij\kappa} = E(x_i x_j x_\kappa^2)$, and the K_{ij} and $K_{ij\kappa}$ would be the corresponding cummulants. These may be extracted in terms of the power series to yield

$$\begin{aligned}
G_\kappa &= K_\kappa \\
G_{\kappa\kappa'} &= K_{\kappa\kappa'} + K_\kappa K_{\kappa'} \\
G_{\kappa\kappa'\kappa''} &= K_{\kappa\kappa'\kappa''} + K_\kappa K_{\kappa'\kappa''} + \\
&\quad K_{\kappa'} K_{\kappa''\kappa} + K_{\kappa''} K_{\kappa\kappa'} + K_\kappa K_{\kappa'} K_{\kappa''} \\
G_{\kappa\kappa'\kappa''\kappa'''} &= K_{\kappa\kappa'\kappa''\kappa'''} + K_\kappa K_{\kappa'\kappa''\kappa'''} + K_{\kappa'} K_{\kappa''\kappa'''\kappa} + \\
&\quad K_{\kappa''} K_{\kappa'''\kappa\kappa'} + K_{\kappa'''} K_{\kappa\kappa'\kappa''} + K_{\kappa'''\kappa'} K_{\kappa''\kappa} + \\
&\quad K_{\kappa'\kappa''} K_{\kappa'''\kappa} + K_{\kappa'''\kappa''} K_{\kappa\kappa'} + 2K_\kappa K_{\kappa'} K_{\kappa''\kappa'''} + \\
&\quad 2K_\kappa K_{\kappa'''} K_{\kappa'\kappa''} + 2K_\kappa K_{\kappa''} K_{\kappa'\kappa'''} + 2K_{\kappa'} K_{\kappa'''} K_{\kappa\kappa''} + \\
&\quad 2K_{\kappa'} K_{\kappa''} K_{\kappa\kappa'''} + 2K_{\kappa'''} K_{\kappa''} K_{\kappa\kappa'} + K_\kappa K_{\kappa'} K_{\kappa''} K_{\kappa'''}
\end{aligned} \tag{2}$$

We apply this factorization to patterns and test significance constructing null hypotheses and variances by shuffling phenotypes.

1.2 Redescription clusters

Subjects $s \in \mathcal{S}$ are described by a list of features $f_i(s)$ indexed by feature labels $i \in \mathcal{F}$. Each feature has an alphabet $\mathcal{A}_i$ so that $f_i(s) \in \mathcal{A}_i$ which is often binary, but could be defined on the reals. Examples of binary features in $\mathcal{F}$ are phenotypes or disease outcomes which would have a continuum alphabet ($\mathcal{A}_{bmi} = \mathbb{R}$).

For a given $a_i \in \mathcal{A}_i$, the set of subjects that have that value is $f_i^{-1}(a_i) \subseteq \mathcal{S}$. So the list of subjects with a disease can be written $f_{PD}^{-1}(1)$. In the case of continuous variables, the selection of sets is according to a threshold, such as the mean $m(f_i(S))$, mapped to 1 if $f_i(s) \geq m(f_i(S))$.

Patterns may be described in terms of conjunctions $i \wedge j$ for $i, j \in \mathcal{F}$ such that $f_{i\wedge j}^{-1}(a_i, a_j) = f_i^{-1}(a_i) \cap f_j^{-1}(a_j)$ for binary a_i, a_j. This definition is extended to include either atomic i, j, such as hypertension or Type II Diabetes, or to any combinations of conjunctions subject to the logical algebra of $\wedge$ (e.g. $(i \wedge j) \wedge (i \wedge k) = i \wedge j \wedge k$ for $i, j, k \in \mathcal{F}$ subject to values a_i, a_j, a_k. So we can specify the Parkinson's disease (PD) subjects with a motor or non-motor symptom such as walking or handwriting as $f_{Walk\wedge PD}^{-1}(Walk = 1, PD = 1)$. Such combinations of conjunctions i that have more or less members $f_i^{-1}(a)$ than expected by chance are called patterns.

Binomial and other tests of the significance of patterns can be dominated by lower-order correlations among the variables in a pattern. Two distinct patterns that yield the same subsets of subjects, e.g. $f_i^{-1}(a) = f_j^{-1}(a)$, are called "redescriptions". If conjunctions yield a form such as $A \cap B = B$, then it may be deduced that $B \subset A$, and the conditions yielding A and B satisfy $b \Rightarrow a$. In other words, redescriptions can reveal logical relationships among features. Such relationships may reflect underlying biological pathways reflected in these connected phenotype patterns. Therefore, each of these patterns i specify a phenotype, which may be associated with genotypes or other -omic data using standard methods.

Given the presence of misclassifications, differential evolution of disease stages, simple transcription mistakes, etc, result in errors in estimates of $f_i^{-1}(a)$ must be accounted for in estimating equivalence. We can use Jaccard distances $d = 1 - \frac{|A\cap B|}{|A\cup B|}$ to measure deviations. So $d(A \cup B, B) = 1 - \frac{|A\cup|}{|B|}$ is 0 if $B \subseteq A$, some non-zero value with any $B \nsubseteq A$. This distance measures the probability that samples drawn from A and B are not shared, which gives an index for whether it would be possible to distinguish disruption due to errors or to distinguish

non-biological pathways vs. biological pathways with error.

1.3 Networks

1.3.1 Ranking nodes in the network

The importance of each node is calculated as an aggregate score defined by the mean of the ranks of different network centrality measures: betweenness centrality (number of unweighted shortest paths between all pairs of nodes in the graph that passes through each node); eigenvector centrality (relative importance of a node as compared to its neighbors); degree (number of edges connected to the node); Voterank (a ranking based on a voting scheme between the neighbors of each node); and information centrality, or current flow closeness (based on effective resistance between nodes in a network). Hence, lower the rank is for a node, it is better.

For example, if a node v_i ranks first in betweenness and eigenvector centrality, but ranks fourth in voterank, degree, and information centrality, then it's aggregated rank would $(1+1+4+4+4)/5 = 2.8$. On the other hand, node v_j might rank second in all of the centrality measures, with an aggregated rank of $(2+2+2+2+2)/5 = 2$. Hence, in the final rank, v_j will rank higher than v_i.

1.4 Simulation studies

`CuNAsim` is a multi-omics simulator integrating phenotypes, genotypes, and gene expression levels. To handle the integration of different omics data we started with a multivariate distribution

$$f(x)d^dx = \sqrt{\frac{\det(A)}{(2\pi)^d}}\exp\left(-\frac{1}{2}(x-\mu)^T A(x-\mu)\right)d^dx \quad (3)$$

Components of x were identified as phenotypic (binary, which may include environmental conditions as well), SNP (pairs of binary alleles, one for each of the chromosome pairs), or gene expression (float). Covariances A^{-1} were specified in terms of $\sigma = A^{-1} = \text{cor}(x, x^T)$ where σ is a matrix representing the covariates of the features, and $\text{cor}(x, x^T)$ is specified to yield correlations among phenotypes, alleles between each pair of chromosomes representing Hardy-Weinberg disequilibrium, and among gene expression levels reflecting co-regulation among pathways. Mappings are applied to these variates to represent expression levels, binary, and genotype data.

Correlations between phenotypes, SNPs, and expression levels reflect interactions, including allele impacts on expression levels, relationships between SNPs, expression levels, and disease/phenotype processes, driven by biological pathways. Offsets μ set quantities such as MAF, case/control proportions, and expression level centers. Binary values were mapped from $I(x_i \geq 0)$. The fraction of cases are $E(I(x_i \geq 0))$. Genotypes were mapped from $I(x_I \geq 0) + I(x_{i+1} \geq 0)$. MAF is then $E(I(x_i \geq 0))$. ORs may be derived from the joint probabilities $E(I(x_i \geq 0 \wedge x_j \geq 0))$ for SNP values x_i. Expression levels were mapped to $\exp(x_i)$.

2 Results

`Remics` was applied on a host of simulation scenarios from `CuNAsim` and six different types of cancer from The Cancer Genome Atlas (TCGA) dataset.

2.1 CuNAsim

Applying `Remics` on a simulation scenario of 1,000 samples with 11 variables (three phenotypes, three SNPs, and five genes). The densely correlated variables were *Gene0, Gene1, SNP1,* and *Gene2*.

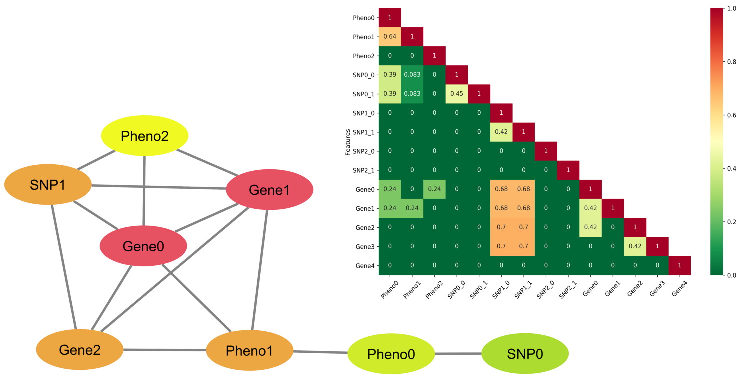

Figure 1: Network of the simulated variables colored by relative importance of each node (darker colors have more importance). The correlation matrix of the variables is shown in the inset with the color gradient.

A more complex simulation scenario with 23 variables (7 genes, 7 phenotypes, and 7 quantitative variables which can represent any omic feature) and 1,000 samples with varying correlations (maximum $r^2 = 0.43$) showed `CuNA` identified a complex network structure reflecting the many but small correlations between variables. *Pheno1* was reflected as the most important node in the network followed by *Quant5, Quant8, and Geno1.* These variables were also densely connected with others in the correlation similarity matrix (Appendix Figure 2).

2.1.1 CuRES

We applied on CuRES on different simulated data (Appendix Figure 3) and observed NRI changing from 1% to 16% based on correlations between variables in the simulated data and their relative association with the target phenotype.

2.2 TCGA data

We found a significant expansion of the feature set when we applied `Remics` to 500 selected features across different types of cancers. The number of significant ($p < 0.01$) redescription groups are shown in Figure 4.

Performing `CuNA` on the statistically significant redescription groups, we obtained the network for each cancer type, with statistically significant edges with the maximum number of edges observed in GBM and the least number being in AML (Figure 5). The number of edges, naturally, is directly proportional to the number of vertices in the network.

2.2.1 Networks

To analyze networks for each cancer type in the TCGA data, we used multiple methods such as centrality analysis, community detection, maximum spanning trees. As these networks are large,

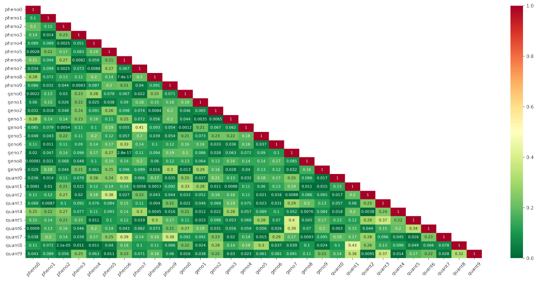

(a) Correlation similarity matrix, simulated from `CuNAsim`.

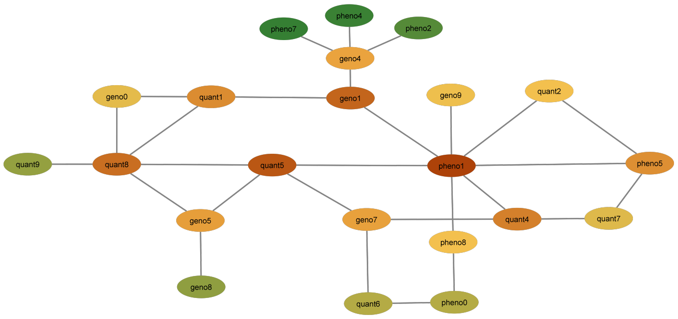

(b) Network of the simulated variables colored by relative importance of each node (darker colors have more importance).

Figure 2: `CuNA` network from a more complex correlated structure among variables.

complex, and dense, to investigate the network for meaningful interactions between multi-omics features we developed an interactive visualizer tool. Using this tool we selected the communities as sub-groups of nodes in the network and studied their interactions within and between communities. Two sub-graphs with neighbors of the queried nodes will be highlighted in two different colors (such as blue and red), respectively, and for overlapping nodes a different colors (such as pink) is used. If one hovers above these nodes or edges, the node importance or edge weight and their label is displayed. This makes it easier to parse complex networks and understand associated biomarkers with a set of selected symptoms, or obtain symptoms related with a genetic target of interest. We discovered connections between ribosomal proteins in OV (Appendix Figure 6), enriched NFE2L2 transcription factor pathway in AML (Appendix Figure 7, DNA methylation markers in GBM (Appendix Figure 8). Interactions between multi-omics features which were validated using the IntAct [1] database of molecular interactions were as follows: {(FGB, LOX), (COL3A1, COL1A1), (COL1A1,LOX), (ITM2A, HBB), (ELANE, HSPA5), (TAGLN, ACTG2)}.

2.3 Complexity Analysis

We used TCGA data to perform a phase transition analyses on a larger data set of 1,000 samples and observed the exponential time and memory required to compute higher-order cumulants. The memory requirement for higher-order cumulants is a greater challenge than the time, as we observe a requirement of 100 GB memory for only 50 features in the dataset (Appendix Figure 10a and 10b).

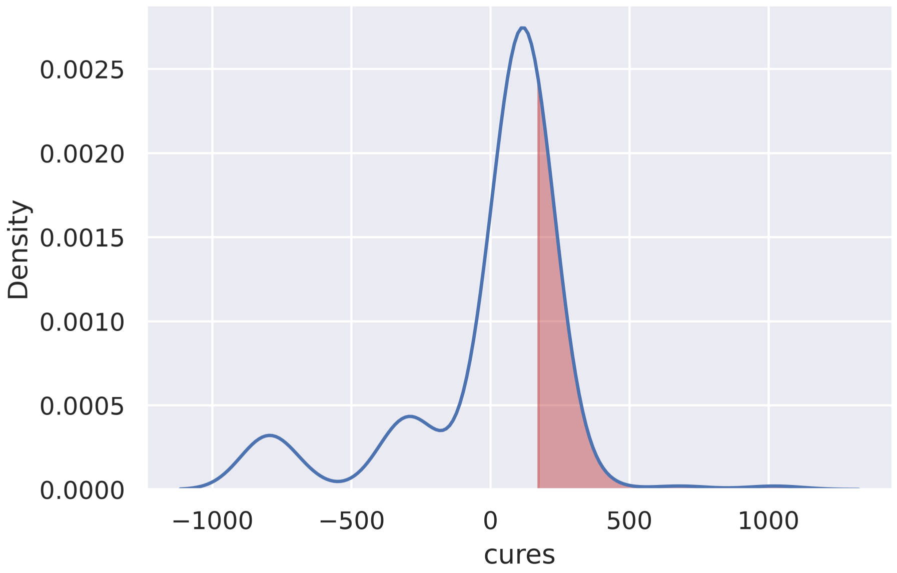

(a) Distribution of CuRES with the top decile colored in red on the simulated data with 1000 samples, 10 variables and target phenotype as *Pheno0*

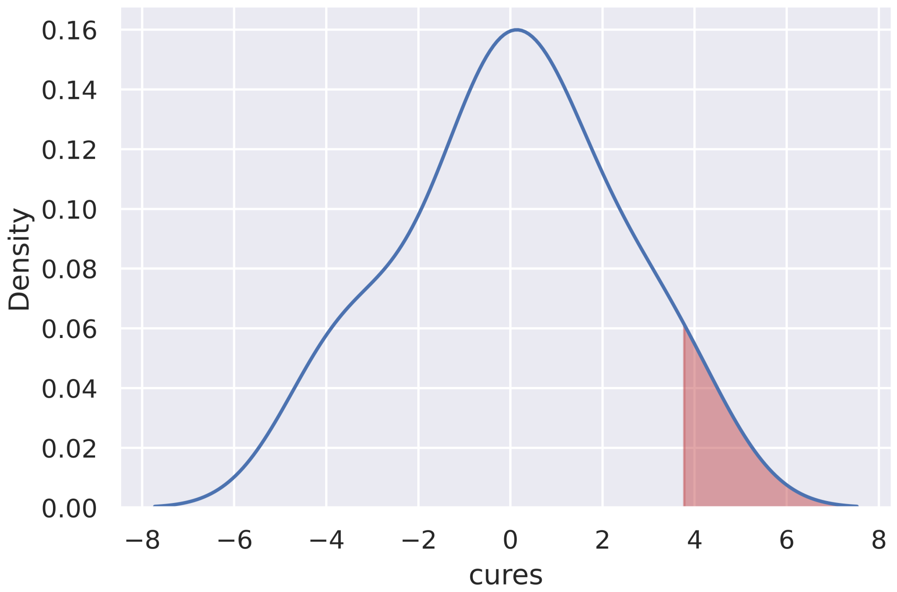

(b) Distribution of CuRES with the top decile colored in red on the simulated data with 1000 samples, 30 variables and target phenotype as *Pheno0*

Figure 3: Distribution of CuRES.

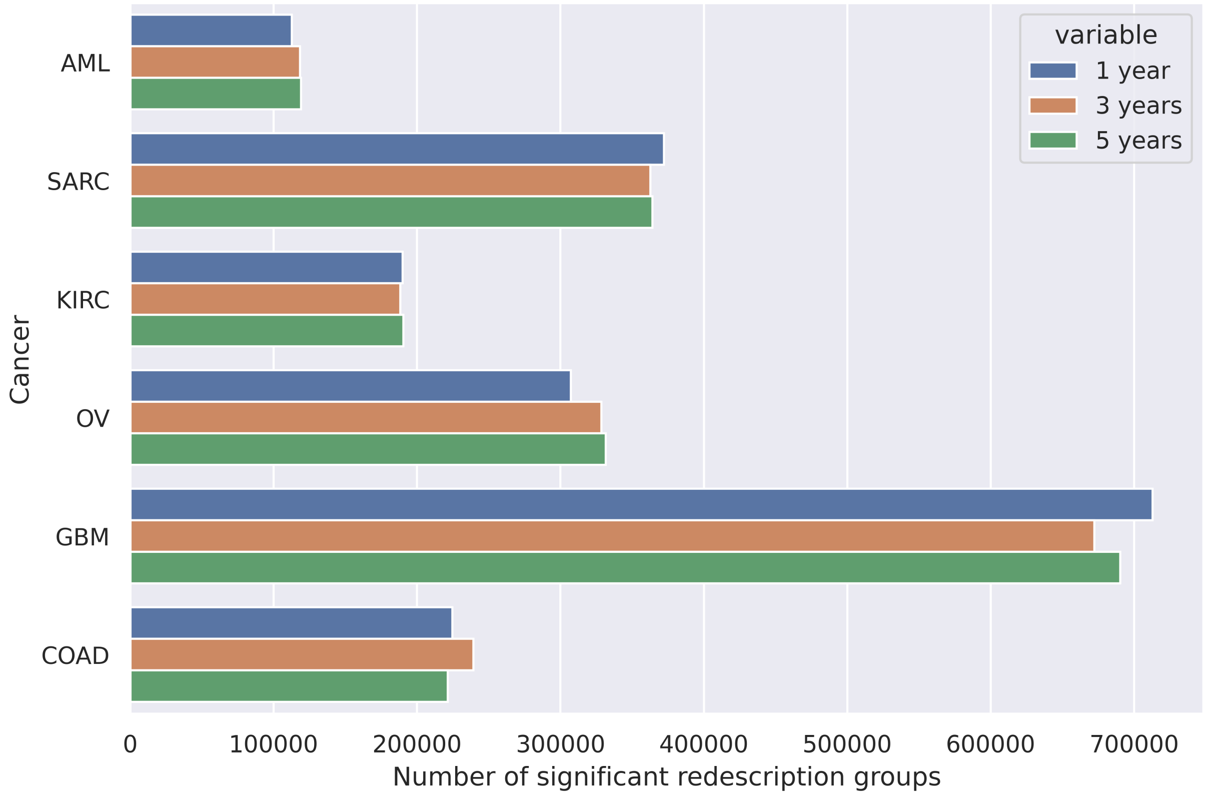

Figure 4: Number of significant redescription groups for each TCGA cancer type considered in the study

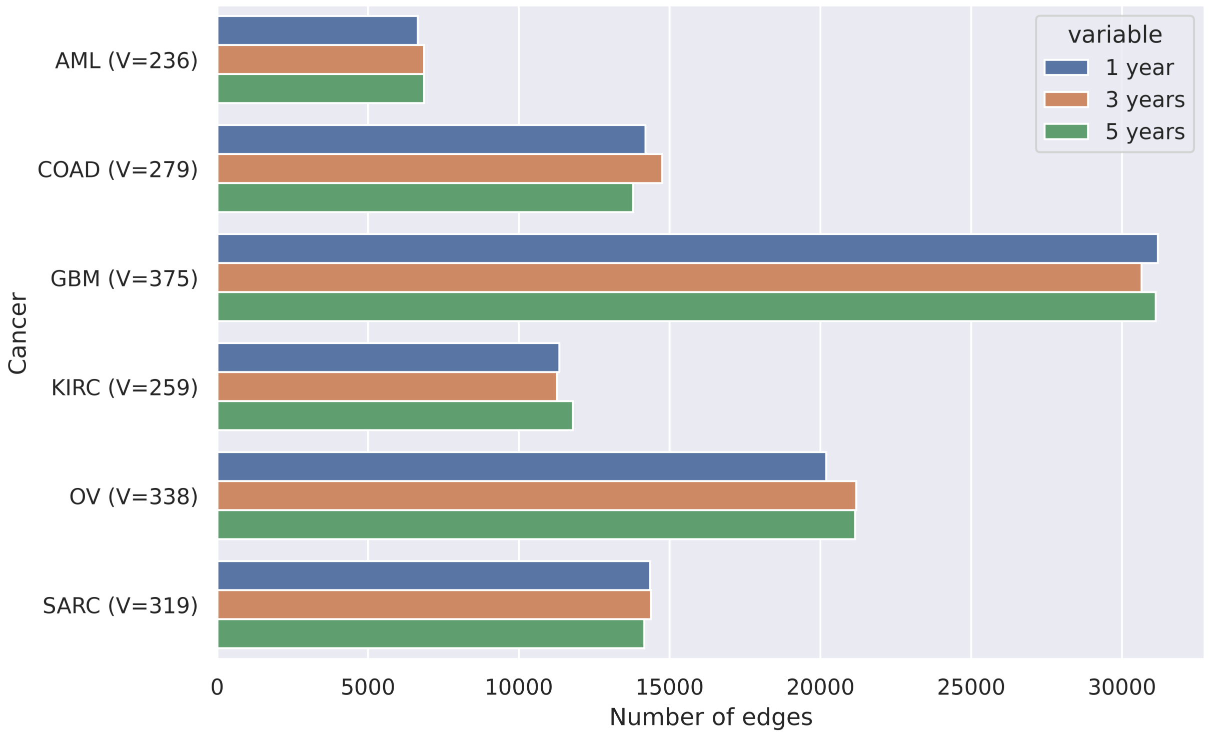

Figure 5: Number of significant edges for each TCGA cancer type considered in the study along with their number of vertices in parenthesis

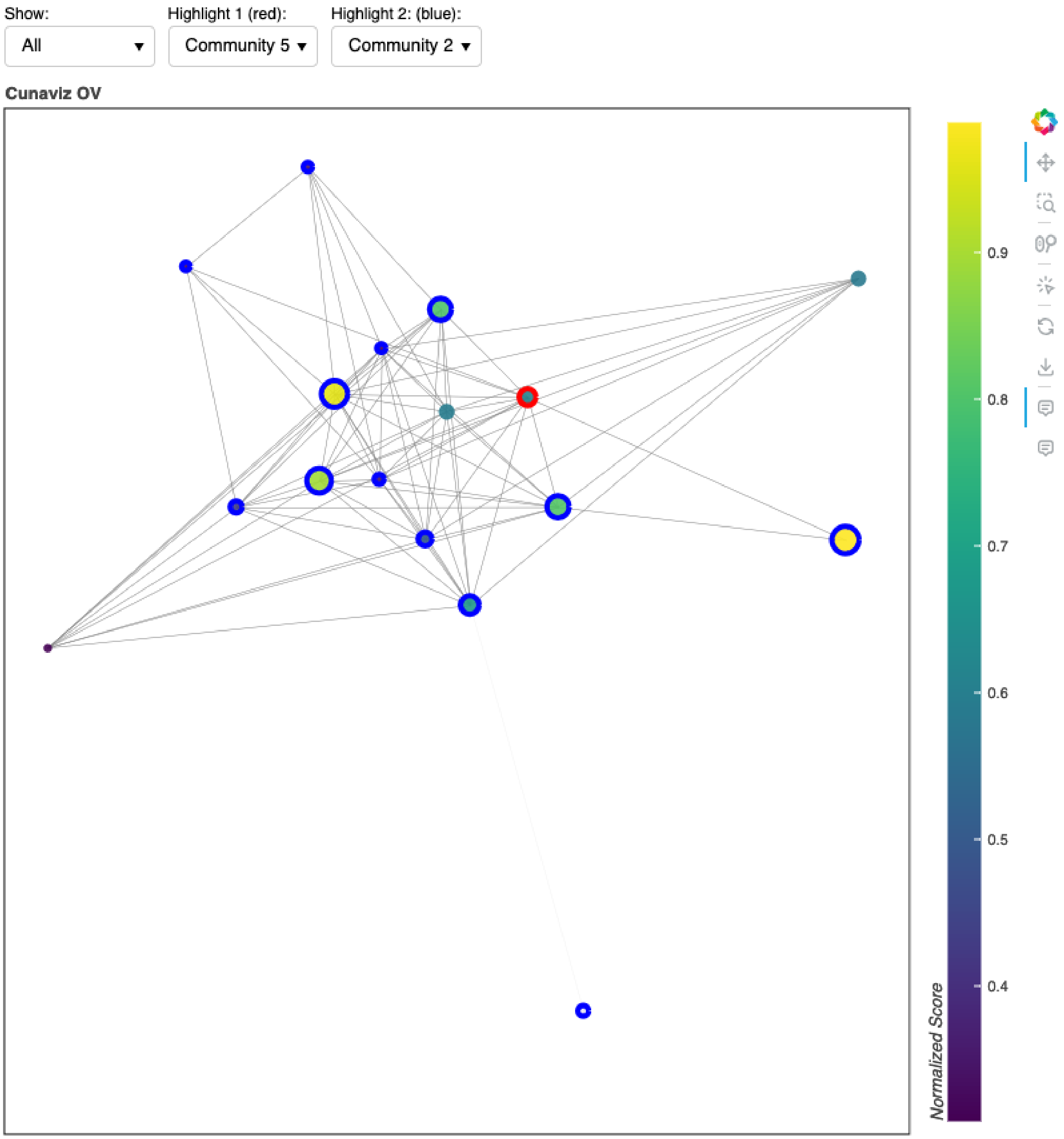

Figure 6: A sub-cluster of vertices in the OV network demonstrating interacting ribosomal protein genes

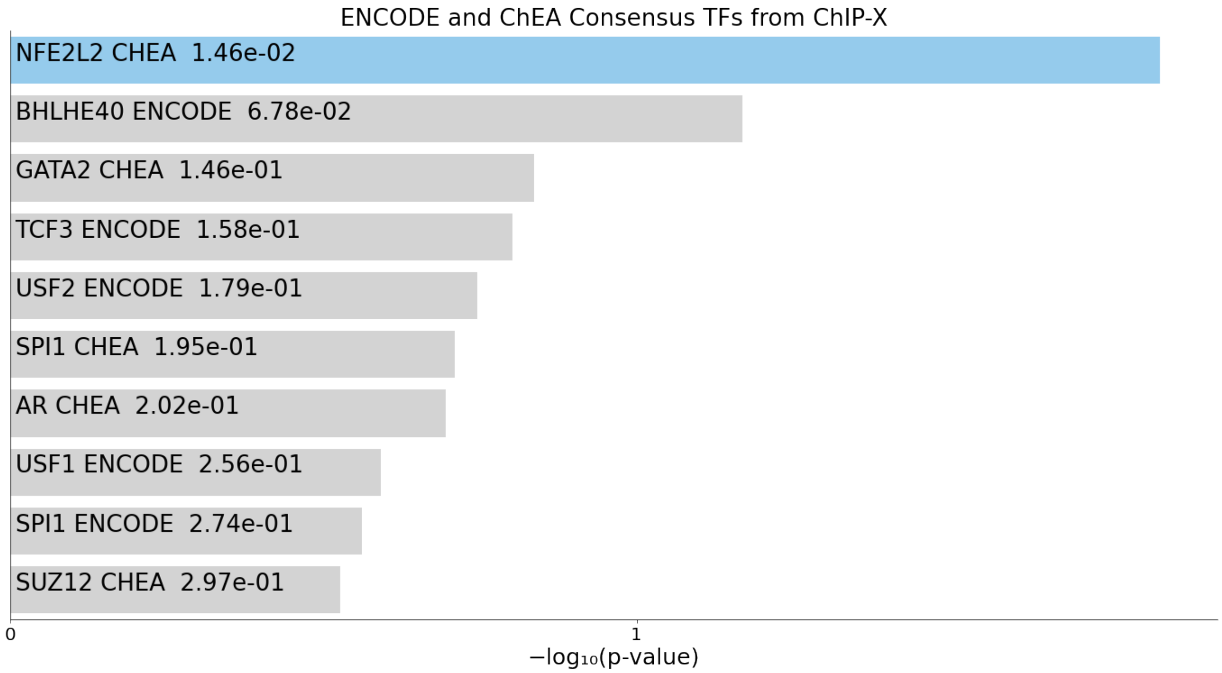

Figure 7: Enriched NFE2L2 pathway in a AML community

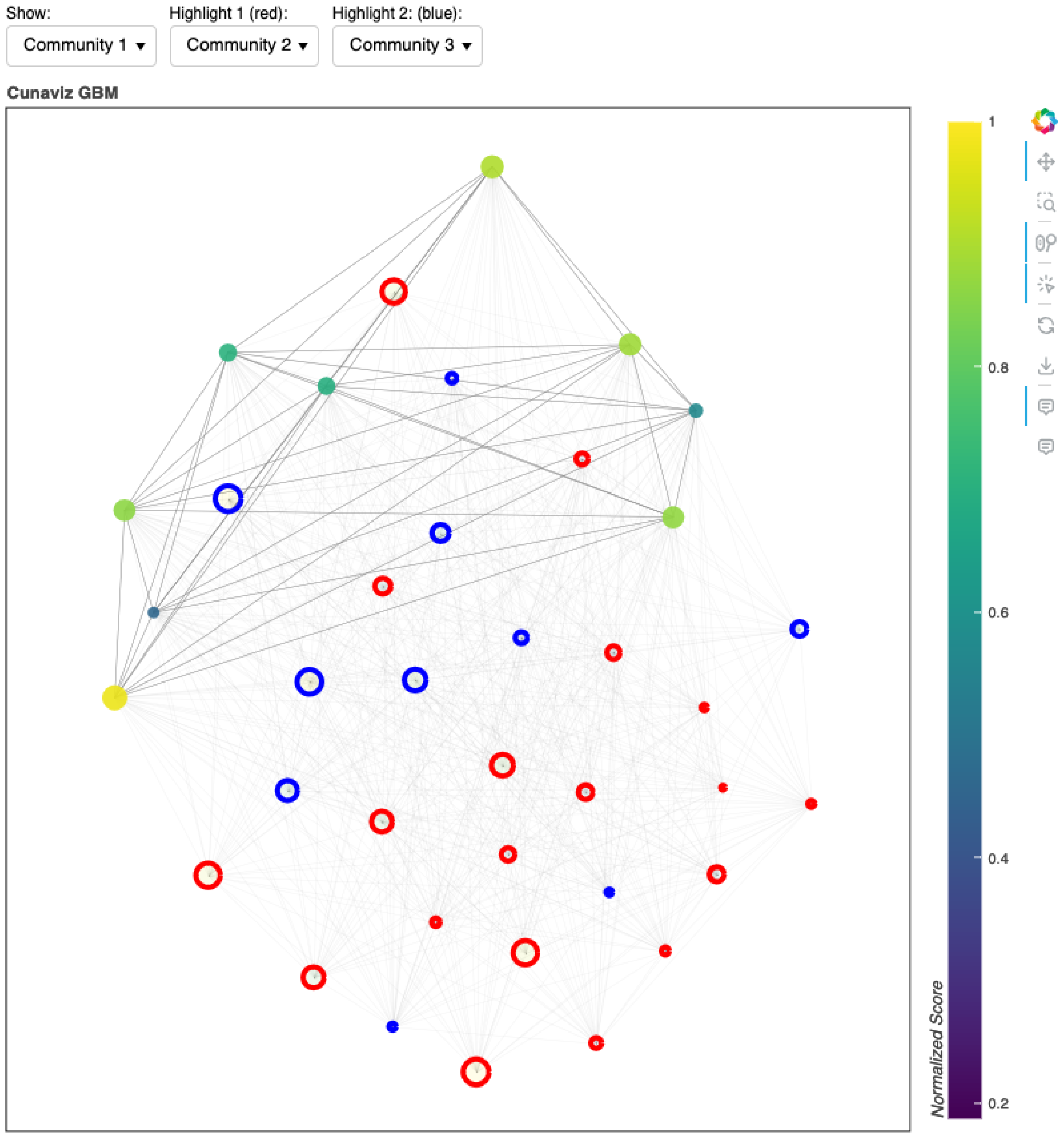

Figure 8: A sub-cluster of vertices in the GBM network demonstrating interacting DNA methylation markers

**Disease**	**Year**	**Data**
AML	1 year	{(hsa-mir-100, STAB1),(RPL13, FTL), (CALR, hsa-mir-100), (ELANE, STAB1)}
	3 years	{(UBC, RPL37), (HSPA5, UBC), (ELANE, STAB1), (HSPA5, RPS11)}
	5 years	{(FTL, UBC), (HERC2P2, CD24), (HSPA5, CTSD), (UBC, RPS2)}
COAD	1 year	{(REG3A, REG1A), (DES, ACTG2), (TAGLN, ACTG2), (MYL9, ACTG2)}
	3 years	{(TAGLN, ACTG2), (ACTG2, FLNA), (TAGLN, FLNA), (DES, MYH11)}
	5 years	{(MYH11, ACTG2), (ACTG2, FLNA), (TAGLN, FLNA), (DES, ACTG2)}
GBM	1 year	{(AKR1B10, HBB), (TSPAN8, SERPINE2), (CHI3L1, C3), (AEBP1, RBP1)}
	3 years	{(AKR1B10, HBB), (KIF1B, IGKC), (STON1, AKR1B10), (IGKC, UCHL1)}
	5 years	{(S100P, ITM2C), (GPR37, CHL1), (hsa-mir-199a*, NMB), (TIMP4, UCHL1)}
KIRC	1 year	{(FGB, CYP1B1), (CYP1B1, LOX), (FGB, CP), (COL3A1, COL1A1)}
	3 years	{(FGB, LOX), (COL3A1, COL1A), (FGB, CP), (hsa-mir-100, SERPINE1)}
	5 years	{(FGB, LOX), (COL3A1, COL1A), (FGB, CP), (IGFBP4, HP)}
OV	1 year	{(DLK1, MEST), (LDHA, hsa-mir-891a), (SRP14, ERBB2), (PYY, GPX3)}
	3 years	{(DLK1, MEST), (hsa-mir-375, DLK1), (TIMP1, RPL18), (RPS8, UQCRFS1)}
	5 years	{(DMKN, LSR), (UQCRFS1, RPS21), (OVGP1, RPS19), (ATP5A1, RPS21)}
SARC	1 year	{(APOD, MPZ), (PYGM, HSP90B1), (GAS6, LAMP1), (TYRP1, CHI3L1)}
	3 years	{(LAMP1, GAS6), (hsa-mir-206, MYH7), (PYGM, HSP90B1), (MPZ, hsa-mir-338)}
	5 years	{(PYGM, HSP90B1), (GAS6, LAMP1), (GPX3, SPP1), (CHI3L1, TYRP1)}

Table 1: Top four edges ranked by their weights in `CuNA` for each cancer type and across all three outcomes

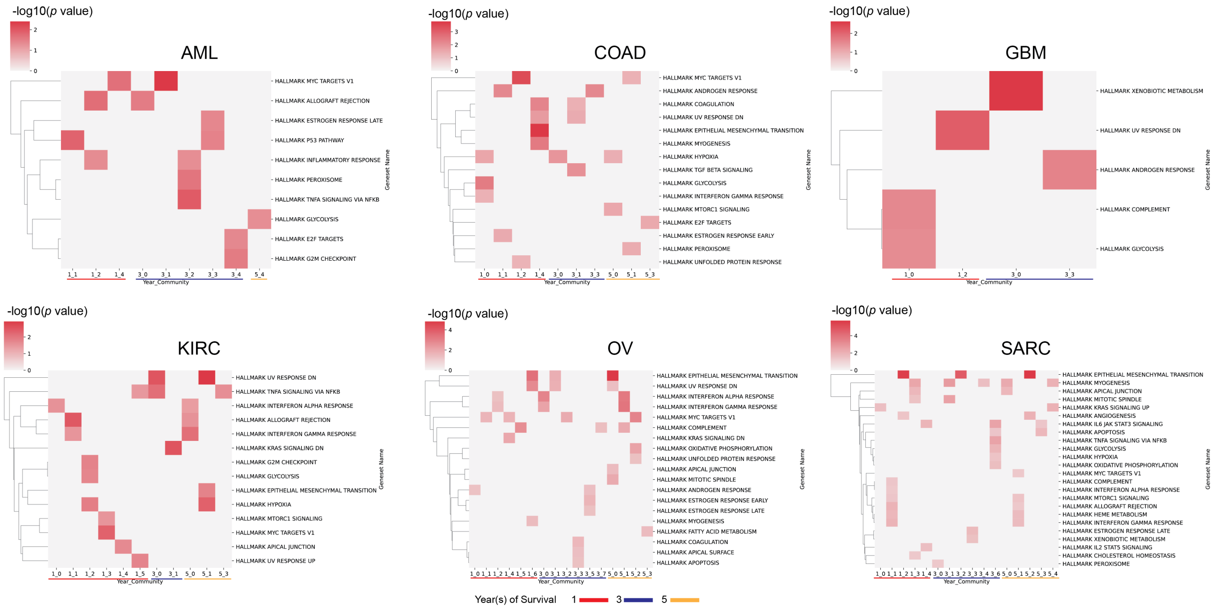

Figure 9: Significantly enriched MSigDB Hallmark geneset in a given community per year of survival per cancer. Significance was determine by a hypergeometric test for enrichment between each Hallmark geneset and the community of interest. The corresponding year(s) of survival the community was detected is denoted in the column name as well as by color where 1-year is red, 3-year is blue, and 5-year is orange.

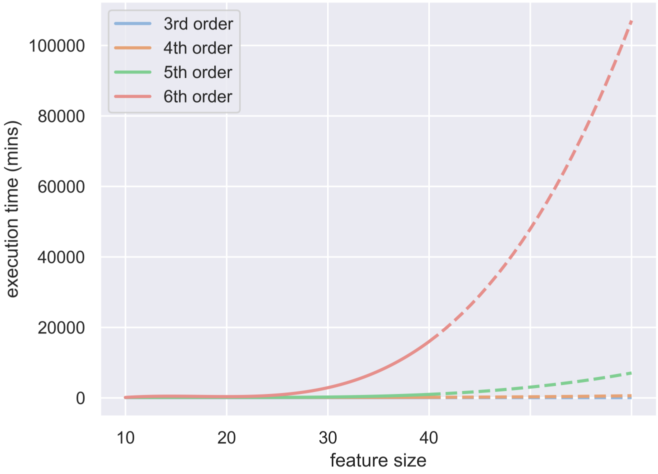

(a) Phase transition of the time required to compute cumulants with varying order of computation

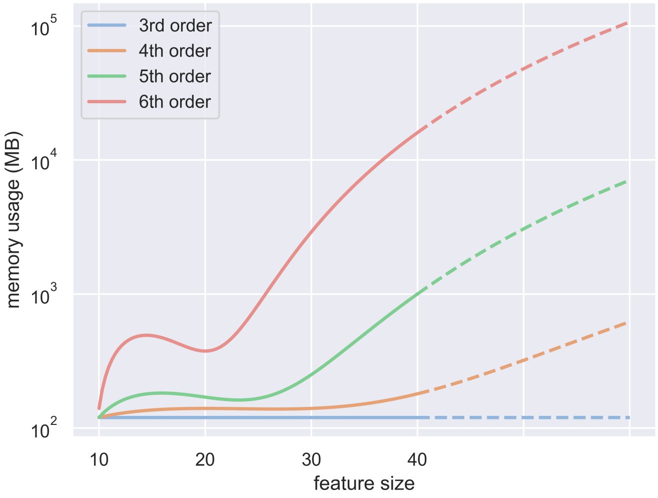

(b) Phase transition of the space required to compute cumulants with varying order of computation

Figure 10: Phase transition of time and memory for TCGA data with 1000 samples

References

[1] Noemi Del Toro, Anjali Shrivastava, Eliot Ragueneau, Birgit Meldal, Colin Combe, Elisabet Barrera, Livia Perfetto, Karyn How, Prashansa Ratan, Gautam Shirodkar, et al. The intact database: efficient access to fine-grained molecular interaction data. *Nucleic acids research*, 50(D1):D648–D653, 2022.

[2] Kerson Huang. *Quantum Field Theory: From Operators to Path Integrals.* Wiley-VCH, Weinheim, 2nd edition edition, April 2010.

[3] Peter McCullagh. *Tensor Methods in Statistics: Second Edition.* Dover Publications, Mineola, New York, revised, updated edition edition, July 2018.

[4] J. K. Percus. Correlation inequalities for Ising spin lattices. *Communications in Mathematical Physics*, 40(3):283–308, October 1975.

[5] Firas Rassoul-agha and Timp Seppalainen. *A Course on Large Deviations With an Introduction to Gibbs Measures.* American Mathematical Society, Providence, Rhode Island, March 2015.
